# Rapid evolutionary responses to insecticide resistance management interventions by the German cockroach (*Blattella germanica* L.)

**DOI:** 10.1038/s41598-019-44296-y

**Published:** 2019-06-05

**Authors:** Mahsa Fardisi, Ameya D. Gondhalekar, Aaron R. Ashbrook, Michael E. Scharf

**Affiliations:** Purdue University, Department of Entomology, West Lafayette, IN 47907 USA

**Keywords:** Experimental evolution, Asthma

## Abstract

The German cockroach (*Blattella germanica* L.) is a worldwide pest that lives exclusively in human environments. *B. germanica* threatens human health by producing asthma-triggering allergens, vectoring pathogenic/antibiotic-resistant microbes, and by contributing to unhealthy indoor environments. While insecticides are essential for reducing cockroach populations and improving health outcomes, insecticide resistance has been a consistent barrier to cockroach control since the 1950s. We conducted seminal field studies to compare three insecticide resistance intervention strategies for cockroaches and evaluated resistance evolution across multiple generations. Using pre-treatment resistance assessment to drive decisions, we found that single active ingredient (AI) treatments can successfully eliminate cockroaches if starting resistance levels are low. We further established that rotation treatments intuitively reduce selection pressure, and are effective when insecticides with no/low resistance are used. We also found that mixture products containing thiamethoxam + λ-cyhalothrin AIs were universally ineffective and highly repellent; and finally, evolution of cross-resistance among AIs is a significant, previously unrealized challenge.

## Introduction

*B. germanica* is a worldwide urban pest species that lives entirely in human settings. *B. germanica* and other cockroaches impact human health through production of asthma and rhinitis-triggering allergens, vectoring of enteric pathogens and by causing psychological stress. Sensitization to cockroach allergens is one of the strongest risk factors for the development of asthma in low-income urban populations worldwide. Most significantly, 85% of inner city homes in the U.S. test positive for cockroach allergens and 60–93% of inner-city children with asthma from different populations are sensitized to cockroaches^1–3^. *B. germanica* produces 11 potent aero-allergens that induce acquired immunity through defined pathways^3,4^, as well as increase risks of virus-induced asthma^5^.

Besides being a vector of human enteric pathogens like *Salmonella*, *Enterococcus* and *E. coli*, *B. germanica* is capable of hosting many other bacterial taxa in its digestive tract, including antibiotic-resistant strains^6–19^. Pathogenic eukaryotic microbes have also been linked to *B. germanica*, i.e., protozoa and fungi^6,13,16,17^. The *B. germanica* microbiome increases in complexity through development and contains 10–70 bacterial taxa depending on life stage^18,19^. All of these bacterial taxa except one (genus *Blattabacterium*) are environmentally acquired. Recent evidence further suggests links between cockroach-vectored bacteria and asthma, i.e., cockroaches contribute to house dust microbiomes, which in turn intensify cockroach-induced asthma^5,20–22^.

Effective cockroach control can reduce allergen loads, asthma morbidity and associated economic costs^2,23,24^. However, insecticide resistance has occurred to every insecticide class introduced for cockroach control since the early 1950s^25–31^. This is because *B. germanica* lives in relatively closed populations^32,33^ which facilitates rapid selection for high-level resistance^28,34^. Cockroach baits have been revolutionary for both controlling cockroaches and reducing pesticide loads in urban housing^24,35^, but baits have not been immune from resistance^29,36,37^. Resistance thus continues to exacerbate impacts of cockroaches on public health. Resistance to more than one class of insecticides (multiple resistance) also appears ubiquitous among cockroach populations, but is difficult to distinguish from true cross-resistance caused by a single mechanism^34,38^. Insecticide resistance mechanisms documented in *B. germanica* include enzymatic detoxification, target site insensitivity, reduced cuticular penetration and behavioral avoidance^28,31,34,38–42^. Insecticide resistance mechanisms have not yet been defined in the *B. germanica* populations tested in this study, but findings presented here provide some initial insights for future characterization.

Proposed strategies for managing resistance in *B. germanica* include rotating between different products or using mixture products with multiple modes of action, rather than using single active ingredient (AI) products with single modes of action. Our study represented a seminal effort to assess trans-generational impacts of different resistance management strategies on resistance evolution in *B. germanica*. Our objectives were to (a) use pre resistance-monitoring data^38^ to make informed insecticide choices, (b) compare three resistance intervention strategies in the field and (c) assess resistance evolution in surviving cockroach field populations. Our findings show clear links between predicted resistance levels and field performance of insecticides, poor efficacy of insecticide deployment strategies on populations with evolved resistance, and unexpected selection of field populations for broad cross-resistance across insecticides. These findings can directly contribute to reducing impacts of cockroaches and pesticide loads in low-income urban housing on a global scale.

## Results

### Population-level impacts of resistance management interventions

**Figure 1:**
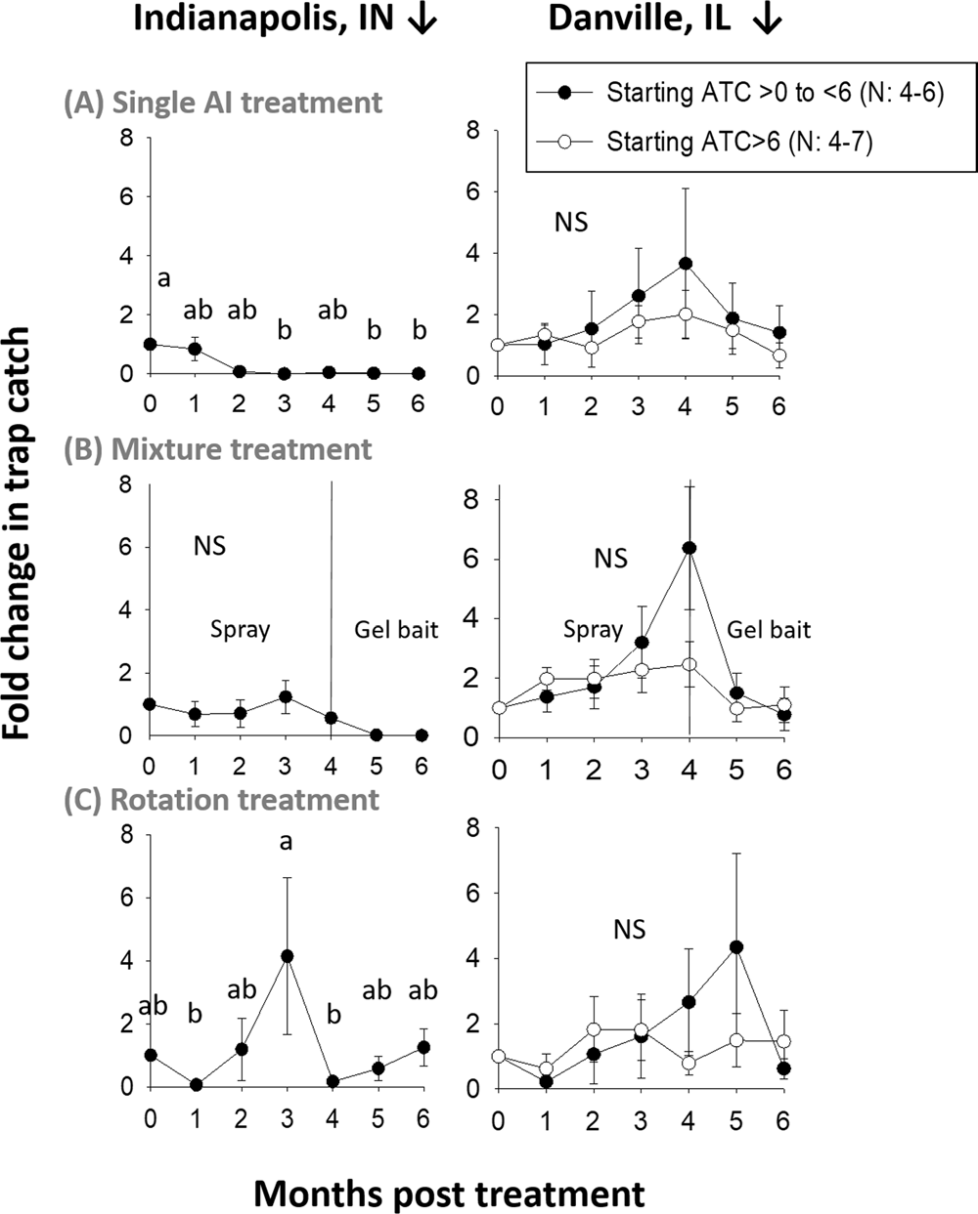
Fold-change in sampled cockroach population numbers in response to different resistance management interventions that consisted of (**A**) a single AI (**B**) mixture product or (**C**) product rotation treatments. See Table 1 for treatment details. The study took place from May (month = 0) to November (month = 6). NS indicates a lack of statistical significance. Numbers (N) of individual apartments per treatment varied from 4–7 per location.

Three resistance intervention approaches were compared in “low-rise” housing facilities in Danville, IL and Indianapolis, IN (in the Midwest USA) that included rotation, mixture or single AI treatments (Table 1). Broad resistance to nearly all available insecticide classes was identified at both study sites based on pre-treatment resistance assessments (RAs) for 14 AIs, including: indoxacarb, abamectin, boric acid, beta-cyfluthrin, bifenthrin, λ-cyhalothrin, fipronil, dinotefuran, imidacloprid, acetamiprid, clothianidin, thiamethoxam, chlorfenapyr and hydramethylnon^38^. Insecticide products with AIs having lowest resistance levels were chosen for application in the field study, i.e., abamectin (Avermectin class; IRAC category 6), boric acid (Inorganic class; IRAC category 8), thiamethoxam (Neonicotinoid class; IRAC category 4). All products were E.P.A. registered, purchased from retail vendors and applied in collaboration with licensed pest management professionals. The study was conducted with human subjects research approval by the Purdue University Institutional Review Board. Untreated control apartments were not permitted, which necessitated comparisons only among the three treatments (i.e., rotation, mixture and single AI). Cockroach population monitoring and density assessment was done with glue traps to determine the amount of insecticide products to apply and to assess treatment impacts. Monthly monitoring results throughout the study are presented for each housing site separately in Fig. 1. Starting trap catches at study initiation were significantly different between the two field sites (One-way ANOVA, F_(1,98)_ = 21.7 P < 0.0001). For this reason, treatment effects were compared only within each location. In general, over the entire study, average numbers of cockroaches per trap per apartment were higher in Danville (mean = 9.8, SE = 1.1) than in Indianapolis (mean = 0.4, SE = 0.08).

**Table 1:** Study design (top) and number of apartments included in resistance intervention strategies (bottom) tested in parallel at two housing sites in Danville, IL and Indianapolis, IN. Abbreviations are as follow: Ab, Abamectin (bait; *Vendetta*); Ba, Boric acid (bait; *Magnetic*); T + λ, Thiamethoxam + λ-cyhalothrin (spray; *Tandem*); Ab + P, Abamectin + Pyriproxyfen (bait; *Vendetta Plus*); C, Collect live cockroaches for lab colony rearing and future resistance assessment (RA). Density categories are based on initial month-0 population assessments from average trap catches (ATC): (i) ATC >0 to <6, (ii) ATC > 6 and (iii) adjacent apartments with starting ATC = 0.

Months post-treatment		Resistance management treatments		
		Single AI	Rotation	Mixture
0	May	Ab	Ab	T + λ
1	June	Ab	Ba	T + λ
2	July	Ab	T + λ	T + λ
3	August	Ab	Ab	T + λ
4	September	Ab	Ba	C + (Ab + P)
5	October	Ab	T + λ	Ab + P
6	November	C	C	No action
**Density category**		**Number of apartments per treatment**		
Indianapolis, IN	i	5	4	5
	ii	—	—	—
	iii	9	15	14
	Total	14	19	19
Danville, IL	i	5	6	4
	ii	4	4	7
	iii	7	8	3
	Total	16	18	14

To account for potential cockroach movement between apartments and buildings, all apartments within study buildings, whether initially infested or not, received identical treatments; and all buildings receiving the same treatments were clustered together. To account for potential density effects on field study results, apartments were divided into three categories based on their starting cockroach population density: (i) Average Trap Catch (ATC) >0 to <6, (ii) ATC > 6 and (iii) adjacent apartments with starting ATC = 0. In Indianapolis, apartments received only “i” and “iii” classifications, while apartments in Danville had all three classifications. Starting cockroach numbers were statistically different among the three categories (Factorial ANOVA, F_(2,39)_ = 28.57 P < 0.0001), but identical across assigned treatments within the Danville site (Factorial ANOVA, F_(treatment×density) (4,39)_ = 0.02 P = 0.999). In Indianapolis, starting cockroach numbers in categories (i) and (iii) varied across treatments (Factorial ANOVA, F_(treatment×density) (2,46)_ = 12.26 P < 0.0001).

**Figure 2:**
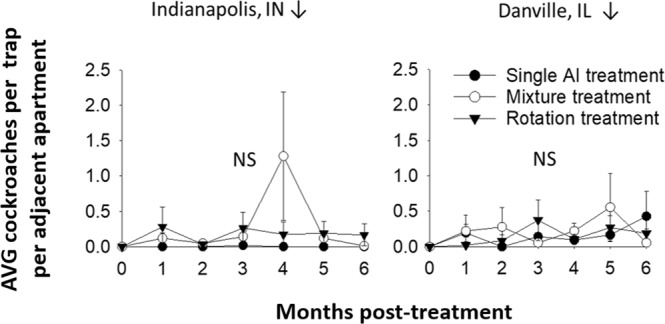
Populations estimates over time for adjacent “category-iii” apartments having 0 cockroaches sampled at study initiation. Results show field trap catches in single AI, mixture and rotation treatments from May (month = 0) to November (month = 6). Numbers of apartments per treatment varied between 9–15 and 3–8 for Indianapolis, IN and Danville, IL respectively. NS indicates a lack of statistical significance.

Treatments were made monthly throughout the 6-month study, immediately after monthly population assessments. Unexpectedly, all treatments failed to reduce ATC to near zero except the single AI (abamectin) treatment in Indianapolis (Fig. 1A–C). In Indianapolis, ATC of apartments in category “i” decreased significantly over the course of the study in single AI treatments and fluctuated in rotation treatments but in mixture treatments fluctuations were non-significant. However, at the Danville site, ATC did not change in any of the treatments over time in either category “i” or “ii” apartments (Table 2, Fig. 1). ATC fluctuated non-significantly in apartment units with low level infestation (category iii), regardless of site or treatment (Fig. 2, Table 2).

**Table 2:** One-way ANOVA results comparing average numbers of cockroaches trapped in field studies at two locations from May to November.

Location	Apartment category^a^	Treatment	F_(df)_	P value
Indianapolis	i	Single AI Treatment	3.83_(6,28)_	0.007
		Mixture Treatment	1.09_(6,28)_	0.4
		Rotation Treatment	2.66_(6,21)_	0.04
	iii	All treatments	0.73_(12,104)_^b^	0.72
Danville	i	Single AI Treatment	0.50_(6,28)_	0.8
		Mixture Treatment	1.24_(6,21)_	0.33
		Rotation Treatment	0.81_(6,34)_	0.57
	ii	Single AI Treatment	0.97_(6,21)_	0.47
		Mixture Treatment	0.70_(6,41)_	0.65
		Rotation Treatment	0.49_(6,21)_	0.81
	iii	All treatments	1.17_(12,242)_^b^	0.31

^a^Categories based on starting trap catches: “i” Average Trap Catch (ATC) >0 to <6, “ii” ATC >6 and “iii” adjacent apartments with starting ATC = 0.

^b^Factorial ANOVA: interaction effect between treatment and time.

Regarding the spray mixture treatment, cockroach numbers increased at both locations despite 4 months of treatment (Fig. 1B). Given the failure of the spray mixture treatment (thiamethoxam + λ-cyhalothrin), our IRB protocol dictated that we rotate to another mixture product after the 4th month, which was a gel bait containing abamectin plus pyriproxyfen (Ab + P; Table 1). Rotating in this manner led to more satisfactory results (after vertical line in Fig. 1B). Cockroaches surviving in treated housing units were live trapped after the 4^th^ month for later resistance monitoring (see below). Overall, the unexpectedly poor performance of a majority of treatments in the field study suggested significant levels of starting resistance and/or selection for higher-level resistance in 4–6 months.

### Resistance evolution to technical active ingredients (AIs)

**Figure 3:**
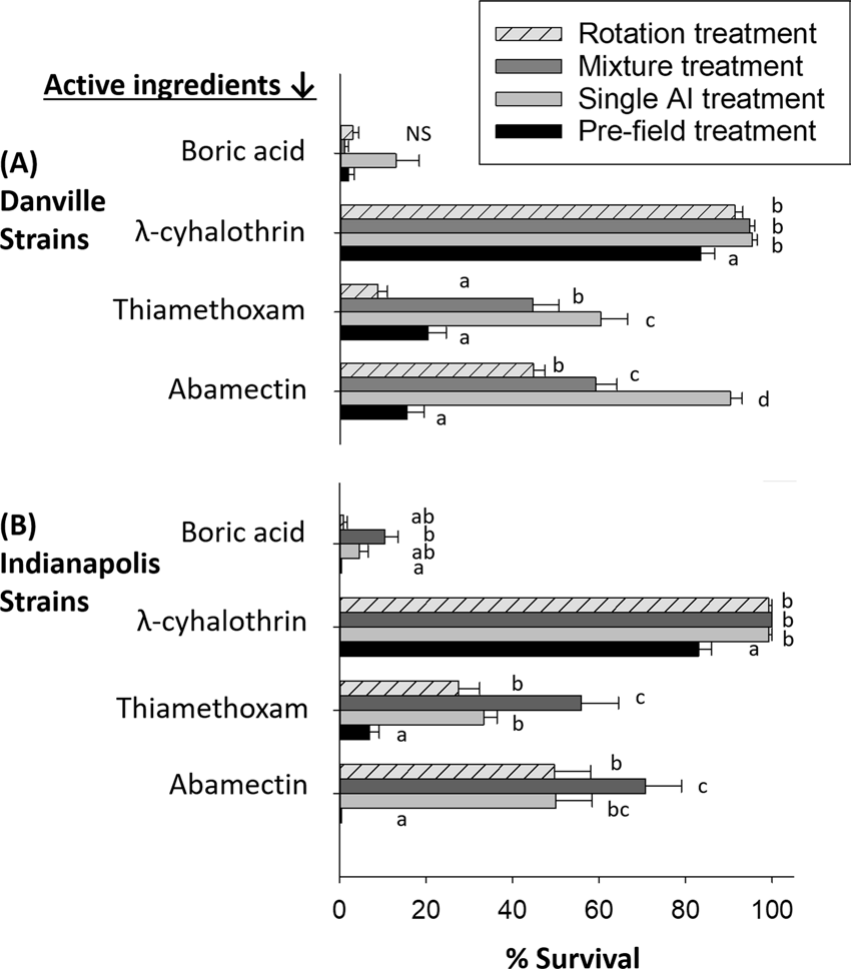
Vial bioassay results showing resistance and cross-resistance evolution in pre- vs. post-treatment cockroach strains from (**A**) Danville IL and (**B**) Indianapolis IN. Assays were conducted by placing lab-reared cockroaches on AI diagnostic concentrations for 24-hr before scoring % survivorship (equivalent to % resistance frequency). For each AI-strain combination, bars with different letters are significantly different (Tukey’s HSD test; P < 0.05). NS indicates a lack of statistical significance between strains. See Table 1 for treatment and AI details. Assay details are provided under *Materials and Methods*.

Based on the above results indicating widespread control failures, we next sought to evaluate physiological resistance and its changes in field-collected populations in responses to insecticide selection pressure. Vials treated with insecticide diagnostic concentrations (DCs), approximating a lethal concentration that killed 99% of a laboratory susceptible strain (JWax-S) (LC_99_)^38^, were used to assess physiological resistance to technical grade AIs. All individuals tested were laboratory-reared and had no prior insecticide exposure. Cockroaches used in DC assays were offspring of individuals either collected at month-4 of the field study (mixture treatment) or month-7 (single AI and rotation treatments). In these assays, percent survivorship as presented (Figs 3, S1) is indicative of the proportion of resistant individuals present in a population. Vial bioassay results revealed statistically significant increases in cockroach resistance frequencies after selection with the majority of AIs applied in the field, regardless of treatment (Figs 3, S1 and Table S1). In contrast, less than 5% survivorship was achieved when the laboratory susceptible strain was exposed to any of the four AIs (i.e., abamectin, thiamethoxam, λ-cyhalothrin and boric acid), which validates the AI-DCs tested.

Bioassays with abamectin indicated selection for higher resistance levels in two generations, as well as unexpected cross-resistance with both AIs of the mixture treatment (thiamethoxam + λ-cyhalothrin) (Figs 3, S1). Specifically, when testing against the abamectin DC, survivorship of field-selected strains collected post-treatment from Indianapolis after all three treatments was significantly increased (50–70.8% increases in survivorship compared with 0% survivorship in pre-treatment strain). The same trend occurred in all three field-selected Danville strains (2.9–5.8 fold increases in survivorship), however, there was already significant 16% resistance present in this population at study initiation. The lack of abamectin resistance in the starting Indianapolis population agrees well with the effective control seen in the field study; whereas, the starting 16% resistance level present in the Danville population was associated with control failures (Fig. 1).

Thiamethoxam bioassays on Indianapolis strains surviving previous exposure to single AI, mixture, and rotation treatments revealed 3.9–8 fold increases in survivorship compared with the pre-treatment strain. In Danville, survivorship increased 2.2–3 fold for strains established after surviving single AI and mixture treatments, while survivorship decreased (0.4 fold change) after the rotation treatment (Fig. 3). Survivorship of field-collected strains assayed with λ-cyhalothrin was more than 94%, and increased 1.1–1.2 fold for strains collected after resistance interventions from Indianapolis and Danville. These results further illustrate the unexpectedly rapid selection for resistance that is possible across diverse AIs.

Conversely, field-collected cockroaches from single AI (abamectin) treatments that were not exposed to the spray AIs, thiamethoxam and λ-cyhalothrin, unexpectedly showed increased cross-resistance to these AIs **(**Fig. 3). Finally, survivorship in boric acid DC assays was less than 13.3% in all post-treatment strains and in all cases but one (Indianapolis-mixture treatment) it was non-significant. From the perspective of selection intensity, abamectin resistance increased the least in the rotation treatment compared with the spray mixture AIs. Likewise, rotation treatments had less impact on thiamethoxam and λ-cyhalothrin resistance selection/evolution; but no impacts on boric acid resistance. Overall, these results support the intuitive idea that AI rotations decrease selection intensity and thus impede resistance evolution.

### Resistance evolution to formulated products (FPs)

**Figure 4:**
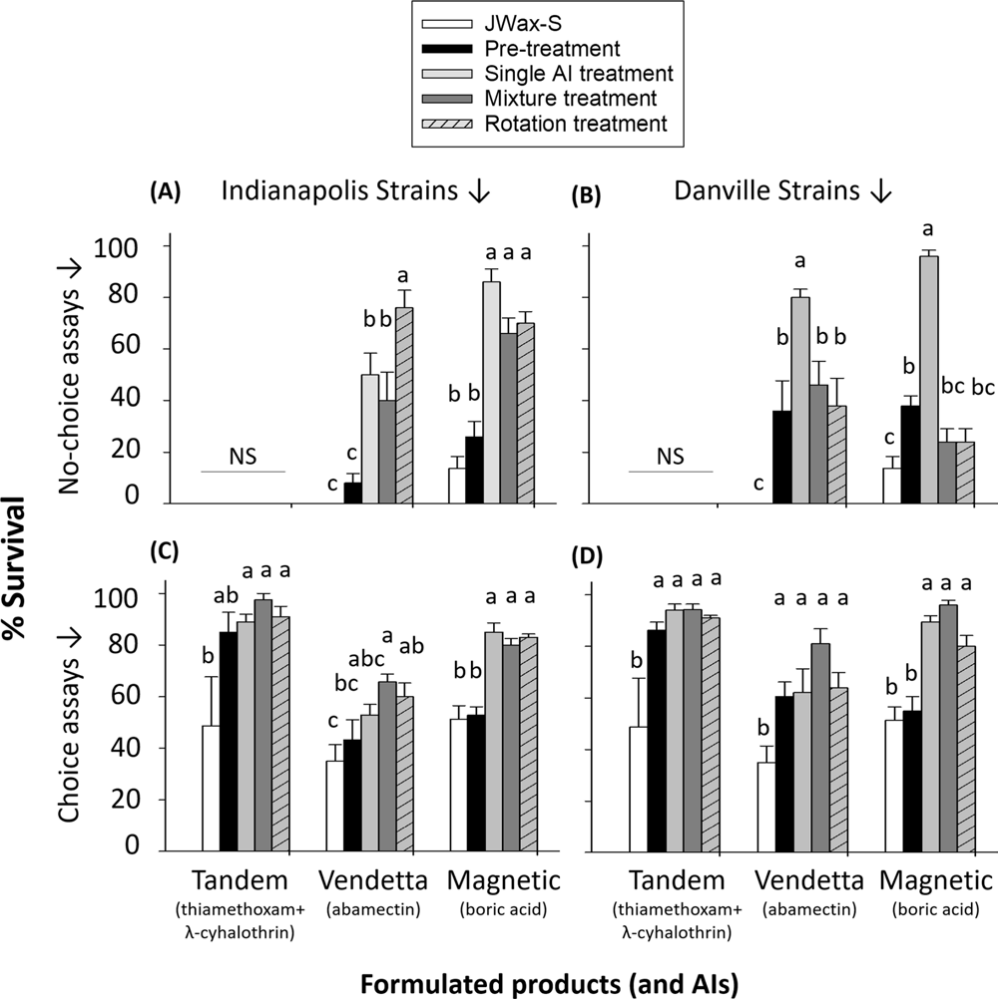
No-choice (**A**,**B**) and choice (**C**,**D**) assay results showing resistance and cross-resistance evolution to formulated products (FPs) in pre- vs. post-treatment cockroach strains from Indianapolis IN (*left*) and Danville IL (*right*). The susceptible JWax-S laboratory strain was included as a reference for calibration purposes. Assays were conducted by holding lab-reared cockroaches with various formulated products for 4-d before scoring survivorship. For each product-strain combination, bars with different letters are significantly different (Tukey’s HSD test; P < 0.05). NS indicates a lack of statistical significance between strains. See Table 1 for treatment and product details. Assay details are provided under *Materials and Methods*.

Based on the above results showing extensive product failures in the field and concurrent evolution of resistance to AIs, we next sought to investigate resistance to the same FPs (purchased from commercial vendors) as used in field studies. First, no-choice bioassays were conducted to screen for physiological resistance (Table S2, Figs 4A,B, S2). Then, choice bioassays were used to investigate behavioral and physiological resistance by simulating field environments in which cockroaches can make behavioral choices and move freely between treated and un-treated areas. When exposed to FPs (Table 1), time-mortality data were scored for 15 d (Figs S2, S3). Lethal time (LT_50_ and LT_90_) values were estimated from these data (Tables S2, S3) but due to (a) rapid insect mortality when exposed to Tandem in no-choice assays, (b) inconsistent resistance ratios (RRs) calculated from Probit analysis results, and (c) an inability to meet all Probit analysis assumptions, we relied mainly on 4-d survivorship data to gain more statistically-supported insights (Table S4, Fig. 4A–D).

Survivorship was generally elevated in choice assays for all field-selected strains regardless of FP (Figs 4C,D, S3). When exposed to Tandem (thiamethoxam + λ-cyhalothrin), 4-d survivorship was significantly lower in laboratory susceptible than field strains (Fig. 4C,D). After 15 d, more than 70% survival was observed in pre-treatment strains collected from both sites, indicating a high level of baseline resistance to this mixture product. Additionally, suvivorship in the susceptible strain never dropped to 0% in choice assays, as compared to 0% survivorship occurring in no-choice bioassays after 2 h for the susceptible strain (and up to 2 d for field strains), indicating significant inherent repellency by this product (Figs 4 and S2, S3a,b). These results further confirm the role of resistance in control failures seen for the mixture treatment in the field study, and further confirm resistance/cross-resistance to AIs revealed by DC screening assays.

When testing the Vendetta product (AI = abamectin), despite there being no survivorship differences in choice assays (Fig. 4C,D), there was increased resistance in no-choice assays for strains collected after surviving the single AI (abamectin) field treatment (Fig. 4A,B). The Indianapolis strain surviving the single AI treament also had lower LT values than the Danville strain (Tables S2, S3), which is consistent with its lower beginning tolerance status at month 0. Furthermore, higher 4-d survivorship and longer LT values for Indianpolis strains surviving the spray mixture treatment, compared with the pre-treatment strain, provide further confirmation of cross-resistance between Vendetta and Tandem AIs. Finally, when exposed to the Magnetic product, 4-d survivorship was generally higher in post-treatment than pre-treatment strains. In choice assays, LT_50_ and LT_90_ values for Magnetic gel bait were longer after selection and ranged between 2.8–18.4 d and 4.2–50.5 d respectively (Table S3, Figs 4, S3). These results explain the increase in field population size in rotation treatments after boric acid bait and mixture product application at both sites.

## Discussion

This study represents a seminal effort to test insecticide resistance intervention strategies for *B. germanica*, which is a medically-significant pest to urban and impoverished populations on a global scale. *B. germanica* is a vector of allergens, human enteric pathogens and antibiotic-resistant bacteria^6–15^. Insecticides have been essential for cockroach management for decades, but resistance has been a barrier to effective control since the 1950s^25–31^. Cockroach baits, widely used in management programs, have been effective for decreasing health impacts of cockroaches on humans and reducing pesticide loads in urban environments^24,35^. Nevertheless, cockroaches can rapidly develop high-level resistance to even bait insecticides^29,36,37^. Minimizing the impacts of insecticide resistance in *B. germanica* using pre-treatment resistance assessments or product rotations, as detailed here, can reduce pesticide loads and cockroach health impacts in urban environments.

This research was focused on testing resistance management strategies in the field (single AI, mixture and rotation), as well as quantifying the rates of resistance evolution and extent of cross-resistance that occurred in response to interventions. Integrated Pest Management (IPM) practices such as sanitation and structural modifications are recommended for use along with baits. However, pest management professionals rely heavily on easy-to-use and cost-effective gel bait-based control strategies. Thus, our insecticide-based field treatment strategies mimicked widely accepted practices followed by the urban pest management industry in public housing. Additionally, our bait use strategy was driven by cockroach population density^43,44^. To add strength to our experimental design, we included two housing sites in the study that were separated by ca. 100 miles. To eliminate potential migration effects, all apartments within study buildings received the same treatments, and all buildings within treatments were clustered together and separated from other treatments by non-study buildings.

All of the tested resistance intervention strategies selected for increased resistance to all AIs applied in field treatments except boric acid. Suprisingly, the single AI treatment (abamectin gel bait) was the only strategy to successfully reduce cockroach numbers. However, these successful reductions only occurred in the study location having low starting resistance to the abamectin AI (Indianapolis), which highlights the importance of pre-treatment RA for choosing effective AIs. Based on broad multi/cross resistance seen in pre-treatment monitoring^38^, AI choices for use in the field were limited. Additionally, the spray mixture treatment not only failed to lower cockroach numbers but also seemed to have a repellent effect that distributed cockroaches to previously uninfested apartments (Fig. 2 months 4–5)^45–47^. This repellency effect was also evident in choice assays with all strains tested, including a susceptible laboratory strain (Fig. 4C,D). These repellency and migration results emphasize a need for targeting as many apartments as possible in infested buildings in order to maximize control efforts. Due to dramatic population increases in the spray mixture treatment, we rotated to a gel bait mixture product containing a combination of abamectin and an insect growth regulator (IGR). Rotating to the abamectin/IGR bait mixture product had a more positive outcome, providing a dual example of both a successful mixture and a rotation.

We also unexpectedly found that the rotation technique, which adds increased mode-of-action diversity and reduced selection pressure on any single AI, was mostly ineffective at reducing cockroach populations due to cross-resistance among AIs. Insecticide cross-resistance has long been known in *B. germanica*^48–50^ and clearly was a factor in the rotation strategy failure in our study. Field results and post-treatment RAs demonstrated cross-resistance between abamectin and two mixture product AIs, thiamethoxam and λ-cyhalothrin, each from different insecticide classes. Although there have been no similar reports of such cross-resistance in cockroaches, cross-resistance to abamectin in pyrethroid-resistant *Musca domestica* and thiamethoxam-resistant *Bemisia tabaci* has been reported^8,51,52^. Our results also showed non-significant, but nonetheless elevated cross-“tolerance” towards boric acid in some instances, suggesting selection for a non-specific resistance mechanism. While cross-resistance profiles among insecticides have been studied for decades, there is a knowledge gap regarding cross-resistance patterns among the broad range of insecticides currently registered for cockroach management. Our findings thus provide important new information on cross-resistance between diverse AIs, as well as insights into the non-specific nature of possible resistance mechanisms. Investigations into possible resistance mechanisms were not part of the current study, but they are ongoing.

Two important limitations of our study are as follows. First is that this study lacked untreated infested-control apartments. Untreated controls were not permitted under the approved IRB protocol, which limited our ability to account for natural seasonal variations in cockroach populations. This limitation was overcome by making comparisons among treatments, and by comparing pre- and post-treatment RAs to document relative impacts of selection intensity on resistance evolution. Our second study limitation is cultural factors having differential impacts on treatment efficacy (i.e., resident behavior, building structure). In terms of resident behavior, our IRB protocol did not permit us to collect resident data and thus we could not evaluate these effects on study outcomes. Our study design instead focused on comparing treatments against each other, which provided novel and valuable insights. Cultural factors do have an impact on structural pest management and should be accounted for in future studies if possible. Alternatively, a potential strength of our study is in its use of FPs that were mass-produced and obtained from commercial vendors. Because of the treatment failures we observed, our findings raise important questions regarding efficacy of mass-produced products.

In this study, based on pre-treatment RA^38^ we chose abamectin, boric acid and thiamethoxam (mixed with λ-cyhalothrin) out of 14 registered and commercially available AIs for cockroach control. However, “over-the-counter” pyrethroid sprays and total release foggers are commonly used by residents against pests, especially cockroaches^53,54^. Our pre-treatment RA showed more than 80% survivorship when cockroaches were exposed to pyrethroid DCs (λ-cyhalothrin, β-cyfluthrin and bifenthrin)^38^, which is consistent with long-standing knowledge of *kdr*-type nerve insensitivity to pyrethroids known in *B. germanica*^34,41,42^. Additionally, after 4 months of exposure to λ-cyhalothrin in the mixture treatment, DC survivorship increased to more than 90% at both housing sites, meaning that the neonicotinoid AI contained in the mixture (thiamethoxam) could not overcome pyrethroid resistance. Pyrethroid residues are the most commonly detected pesticide residues in low-income public and private housing^55,56^. High resistance to pyrethroids in *B. germanica* and their ubiquitous presence in urban housing environments raise concerns regarding the continued unchecked usage of pyrethroids (and total release foggers)^54^ in these settings.

Regardless of resistance intervention strategy, resistance levels increased to almost all AIs tested in this study, with the rotation strategy (based on AI screening) seeming to produce the lowest selection intensity. We also demonstrated here that pre-treatment RA, because of its ability to identify AIs with the lowest resistance, is vital for treatment success. Specifically, we found that single AI treatments can provide control in the short-term when starting resistance levels are low. The best management approach in low-income multi-family housing settings appears to be conducting pre-treatment RA whenever possible, particularly where there is a record of control failure. However, as also shown here and previously^28,34,37^, resistance levels essentially increase with every exposure in direct response to selection pressure placed on closed populations. Rotations thus are essential for minimizing selection pressure in the long-term. In addition, non-chemical IPM approaches that reduce reliance on insecticides would unquestionably provide added benefits^53,57^.

In conclusion, gaining knowledge of cross-resistance patterns between and within insecticide classes is important for designing rotation strategies, and will improve predictability of long-term cockroach management programs. Rotation is a viable recommendation for cockroach resistance management but its success ultimately depends on having low cross-resistance profiles among AIs included in the rotation. Mixture products of all types have potential utility as well, if no cross-resistance between AIs exists and the mixtures are included in rotations. Finally, among all the AIs tested in the field, those in the pyrethroid class were most significantly and uniformly impacted by resistance. This ubiquitous pyrethroid resistance is likely due in part to widespread availability of “over-the-counter” consumer products, and thus, discouraging use of professional/restricted-use pyrethroid products seems a logical measure in federal low-income housing scenarios.

## Materials and Methods

### Cockroach field collection and lab rearing

**Figure 5:**
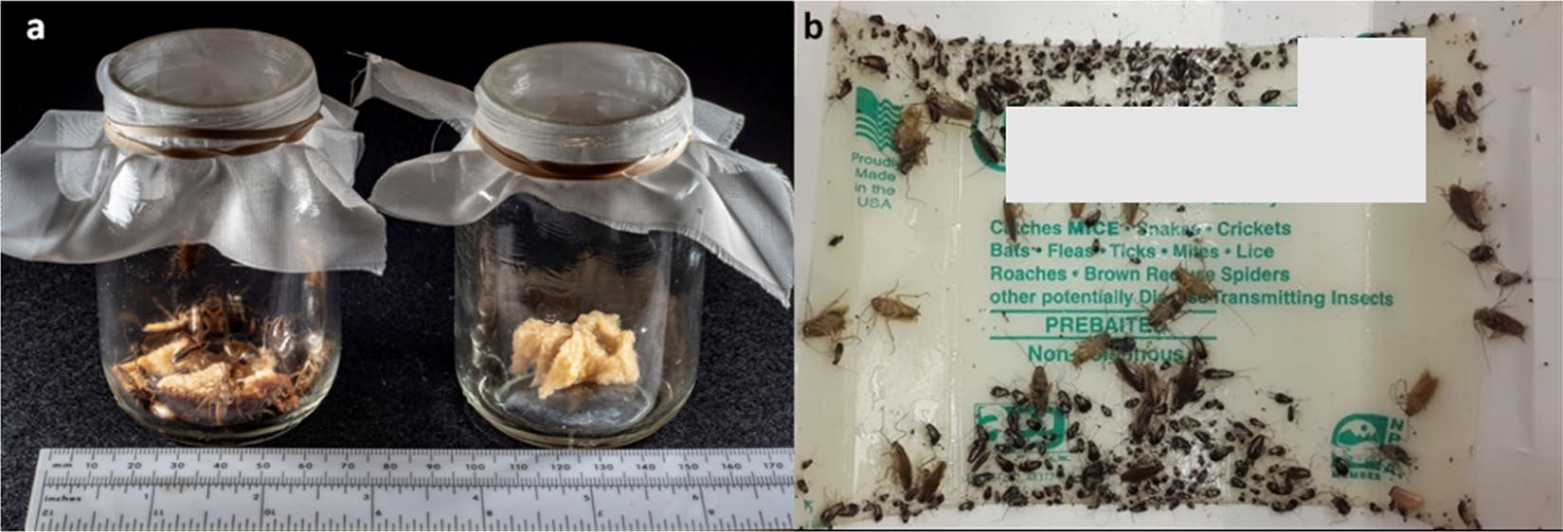
(**a**) Baby food jars used to live-trap *B. germanica* in the field, baited with white bread soaked in beer and greased around the top to prevent escape. (**b**) An example sticky trap used for monitoring cockroach population changes during the field study (product information redacted). Photo credit: John Obermeyer.

*B. germanica* strains were live-trapped using baby food jars (~250 mL), baited with white bread soaked in beer and greased around the top to prevent escape (Fig. 5a). Before field treatment, cockroach strains were collected from multiple apartments in housing sites from Indianapolis, IN and Danville, IL, to establish 2 laboratory “meta” populations. Post-treatment populations were established individually according to location and treatment. The backgrounds of the susceptible strain, JWax-S, and pre-treatment field strains were described previously^38^. Colonies were reared in Ziploc plastic containers (44.3 × 30 × 17 cm^3^/15.14 liter) (IRIS USA, Inc.) with screened lids and held in a controlled-environmental chamber at 26 ± 1 °C and a photoperiod of 12:12 h light:dark, without insecticide selection pressure. Cardboard for shelter, rodent diet (#8604; Harlan Teklad, Madison, WI) and water were provided ad libitum.

### Field monitoring and treatments

Trap catch was recorded monthly as “0” (pre) in May and “1–6” months post-treatment from June to November, 2016. Six non-scented sticky traps (20.3 × 12.7 cm^2^; Mouse and Insect Glue Trap: Catchmaster, AP&G Co., Inc., Bayonne, NJ; purchased from Univar Co. Indianapolis, IN) (Fig. 5b) were placed per apartment at each visit. Trap placements were above and below the kitchen sink, behind the refrigerator, under the oven, in the utility closet/laundry room, and in the bathroom. Formulated products were purchased from Univar Co. (Indianapolis, IN) and included: Vendetta (abamectin B1 0.05%; McLaughlin Gormely King Co., MN, USA), Magnetic (boric acid 33.3%; Nisus Co., TN, USA), Vendetta Plus (abamectin 0.05% + pyriproxyfen 0.5%; McLaughlin Gormely King Co., MN, USA) and Tandem (thiamethoxam 11.6% + λ-cyhalothrin 3.5%; Syngenta Crop Protection, NC, USA). Bait guns and AccuSpray Professional sprayers (B&G Equipment Company, Jackson, GA) were used for crack and crevice treatments in kitchens, utility rooms and bathrooms following label guidelines. Monthly treatments and numbers of apartments per treatment are summarized in Table 1. Tandem spray solution was prepared on-site by mixing 32 ml of Tandem per 3.8 Liter of water (1 gallon) to achieve 0.13% concentration of AIs in the final solution. Old bait residues were removed from apartments in May before study initiation. The amounts of bait and spray applied per apartment are summarized in Table S5.

### Insecticide resistance bioassays

RA bioassays were done using three methods: vial, no-choice, and choice bioassays. Vial and no-choice assays were described previously^38^. For conducting vial bioassays, AIs of >95% purity were purchased from Fisher Scientific (Pittsburgh, PA) or Sigma-Aldrich (St. Louis, MO), and included: abamectin (98.3%), boric acid (99.9%), λ-cyhalothrin (99.5%) and thiamethoxam (99.5%). FPs, used in no-choice and choice bioassays are listed above in the “*Field Monitoring and Treatments*” section. A minimum of 10 replications were performed for vial bioassay and 3–5 replicates for no-choice assays. The only modification is the use of unaged insects for no-choice tests. Choice bioassays were modeled after Ebeling *et al*.^45–47^ using disposable Tupperware^TM^ plastic boxes. Two 6” high x 9” wide plastic boxes, one painted black, were connected near the top by a 1” length of ¼” tubing. One box was treated with the labeled rate of formulated spray or received 0.5-gram of gel bait. The other side (light side) contained only food and water and was not painted. Choice bioassays were held under laboratory conditions and a photoperiod of 24:0 h light:dark. Cockroaches (10 Nymphs, 5 ♂ & ♀ adults each) were released in the light side for acclimation 1 d before the experiment started. Mortality was scored every 2 h and daily after 12 h up to 15 d. To prevent escape, container walls were lightly greased 1” from the top and closed tightly with lids. Only the lid for the light side contained a central meshed opening (3 cm diam). Three to ten replications were done for choice assays.

### Statistical analysis

Two “low-rise” housing sites, located in Indianapolis, IN and Danville, IL were included in the study. At each site, there were 14–19 apartments per treatment, but these were broken down into different density categories^44,58^ for analysis (n = 3–15 per density category; Table 1). Monthly ATC (avg. trap catch) from each apartment was recorded as a single replicate. Analysis of variance (ANOVA) was used for comparing variations in ATC across treatments and time. Post hoc Tukey’s HSD tests were used for mean separations. ATC was analyzed by location due to differences in initial cockroach density between the two study sites. For each location, variation in initial trap catch in May (density) between treatments was compared by two-way ANOVA. Then, monthly ATC was compared within each treatment for category i (ATC > 0 to <6) or ii (ATC > 6) independently. However, in category iii (starting ATC = 0), variation in ATC was compared between the three treatments. Variation in cockroach survivorship obtained from vial, no-choice or choice bioassays between susceptible, pre- and post-treatment strains for each AI or FP was analyzed by ANOVA followed by post hoc Tukey’s HSD test for mean separations for each location. LT values and their corresponding 95% fiducial limits (FLs) were estimated by analyzing time–mortality data using the PROC PROBIT function in SAS 9.4. Control mortality was accounted for in probit analysis. The statistical method described by Robertson *et al*.^59^ was used to estimate RR at LT_50_ and LT_90_. RRs calculated for post-treatment strains were considered significant if the confidence intervals (CI) did not include 1.0.

## Supplementary information

Supplementary Materials

## Data Availability

All data needed to evaluate the conclusions in the paper are present in the paper and/or the Supplementary Materials. Additional data related to this paper may be requested from the authors.
