# Supplementary material for: Rapid evolutionary responses to insecticide resistance management interventions by the German cockroach (Blattella germanica L.)
Source: Sci Rep. 2019 Jun 5;9:8292. doi: 10.1038/s41598-019-44296-y (PMC6549143; doi:10.1038/s41598-019-44296-y)
Supplement: Supplementary file 1 — Supplementary Materials [file 41598_2019_44296_MOESM1_ESM.docx]

**Rapid evolutionary responses to insecticide resistance management interventions by the German cockroach (*Blattella germanica* L.)**

Mahsa Fardisi, Ameya D. Gondhalekar, Aaron R. Ashbrook, Michael E. Scharf*

Purdue University, Department of Entomology, West Lafayette, IN, 47907 USA

*Author for Correspondence:

Email: [mscharf@purdue.edu](mailto:mscharf@purdue.edu), Phone: 765-496-6710

Author contributions: M.E.S. and A.D.G. designed the research; M.F., A.D.G., A.R.A. and M.E.S. conducted the research; M.F. analyzed the data; M.F., A.D.G., A.R.A. and M.E.S. wrote and revised the manuscript.

Supplementary Materials

**Table S1.** One-way ANOVA results indicated for each AI, comparing the effect of treatment strategy (rotation, mixture and single AI treatments) on cockroaches collected before and after field treatments by using diagnostic vial bioassays.

| Location | Active Ingredients | F_(df)_ | P value |
| --- | --- | --- | --- |
| Indianapolis, IN | Abamectin | 83.75_(9, 132)_ | <0.0001 |
|  | Boric acid | 480.73_(9, 99)_ | <0.0001 |
|  | Thiamethoxam | 208.41_(9, 121)_ | <0.0001 |
|  | λ-cyhalothrin | 528.48_(9, 108)_ | <0.0001 |
| Danville, IL | Abamectin | 171.96_(9, 147)_ | <0.0001 |
|  | Boric acid | 320.04_(9, 104)_ | <0.0001 |
|  | Thiamethoxam | 190.56_(9, 115)_ | <0.0001 |
|  | λ-cyhalothrin | 482.35_(9, 114)_ | <0.0001 |

**Table S2**. No-choice assay lethal time (LT) estimation and resulting resistance ratios. Gel baits and spray mixture product lethal time estimates for Vendetta^TM^ gel bait, Magnetic^TM^ gel bait or Tandem® spray tested in a no-chice bioassays against suceptible lab strain (JWax-S), pre- and post-treatment field strains. LT values for post-treatment strains were higher than pre-treatment strains collected from both sites except the highlighted cases.

^1^For each gel bait, TR_50_ values with * are significantly different based on non–overlap of 95% confidence interval. NS indicates a lack of statistical significance between results of pre- and post treatment collected field strains.

^2^FL stands for fiducial limit.

^3^CI stands for confidence interval.

**Table S3.** Choice assay lethal time (LT) estimation and resulting resistance ratios. Gel baits and spray mixture product lethal time estimates for Vendetta^TM^ gel bait, Magnetic^TM^ gel bait or Tandem® spray tested in a choice-box bioassays against a suceptible lab strain (JWax-S), and pre- and post-treatment field strains. LT values for post-treatment strains were higher than pre-treatment strains collected from both sites except the highlighted cases.

^1^For each gel bait, TR_50_ values with * are significantly different based on non–overlap of 95% confidence interval. NS indicates a lack of statistical significance between results of pre- and post treatment collected field strains.

^2^FL stands for fiducial limit.

^3^CI stands for confidence interval.

**Table S4.** One-way ANOVA results indicated for each FP, comparing 4-d mortality on cockroaches collected pre- and post-field treatments by using no-choice and choice bioassays.

| Bioassay | Location | FP | F_(df)_ | P value |
| --- | --- | --- | --- | --- |
| No-choice assay | Indianapolis | Tandem® | 281.2_(9,20)_ | <0.0001 |
|  |  | Vendetta^TM^ | 73.0_(9,46)_ | <0.0001 |
|  |  | Magnetic^TM^ | 66.1_(9,46)_ | <0.0001 |
|  | Danville | Tandem® | 166.1_(9,20)_ | <0.0001 |
|  |  | Vendetta^TM^ | 43.8_(9,46)_ | <0.0001 |
|  |  | Magnetic^TM^ | 92.8_(9,46)_ | <0.0001 |
| Choice assay | Indianapolis | Tandem® | 4.4_(9,26)_ | 0.0015 |
|  |  | Vendetta^TM^ | 36.0_(9,69)_ | <0.0001 |
|  |  | Magnetic^TM^ | 34.2_(9,81)_ | <0.0001 |
|  | Danville | Tandem® | 8.2_(9,41)_ | <0.0001 |
|  |  | Vendetta^TM^ | 25.4_(9,65)_ | <0.0001 |
|  |  | Magnetic^TM^ | 26.4_(9,74)_ | <0.0001 |

**Table S5.** Amount of insecticides applied monthly per apartment based on numbers of cockroaches sampled in trap catches.

| # of cockroaches trapped | Insecticide applied |
| --- | --- |
|  | Gel bait tube (g)/Spray mixture bottle (ml) |
| 0-10 | 7.5/118 |
| 11-50 | 15/236 |
| 51-100 | 30/473 |
| >200 | 60-90/947-1419 |


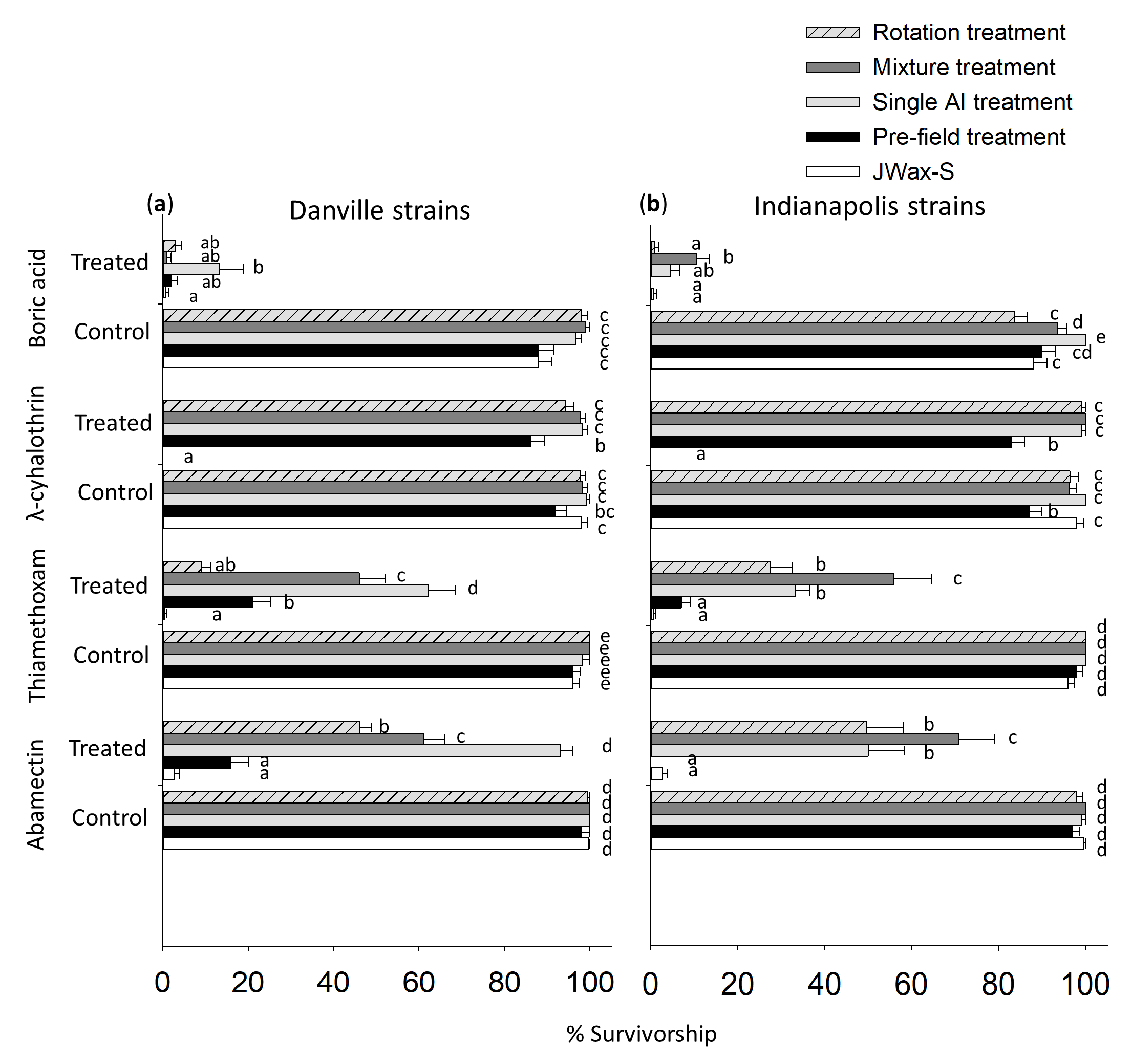


**Fig. S1.** Expanded vial bioassay results showing resistance and cross-resistance profiles in pre- and post-treatment *B. germanica* lab-reared field strains when exposed to AIs at their respective diagnostic concentrations, compared with control treatments. Statistical analysis results (Tukey’s HSD test; P<0.05) are shown for each AI comparing the JWax-S (lab susceptible strain), vs. pre- and post-treatment (**a**) Danville and (**b**) Indianapolis strains. For each AI, strains (shown as bars) with different letters are significantly different. See **Table 1** for treatment details.


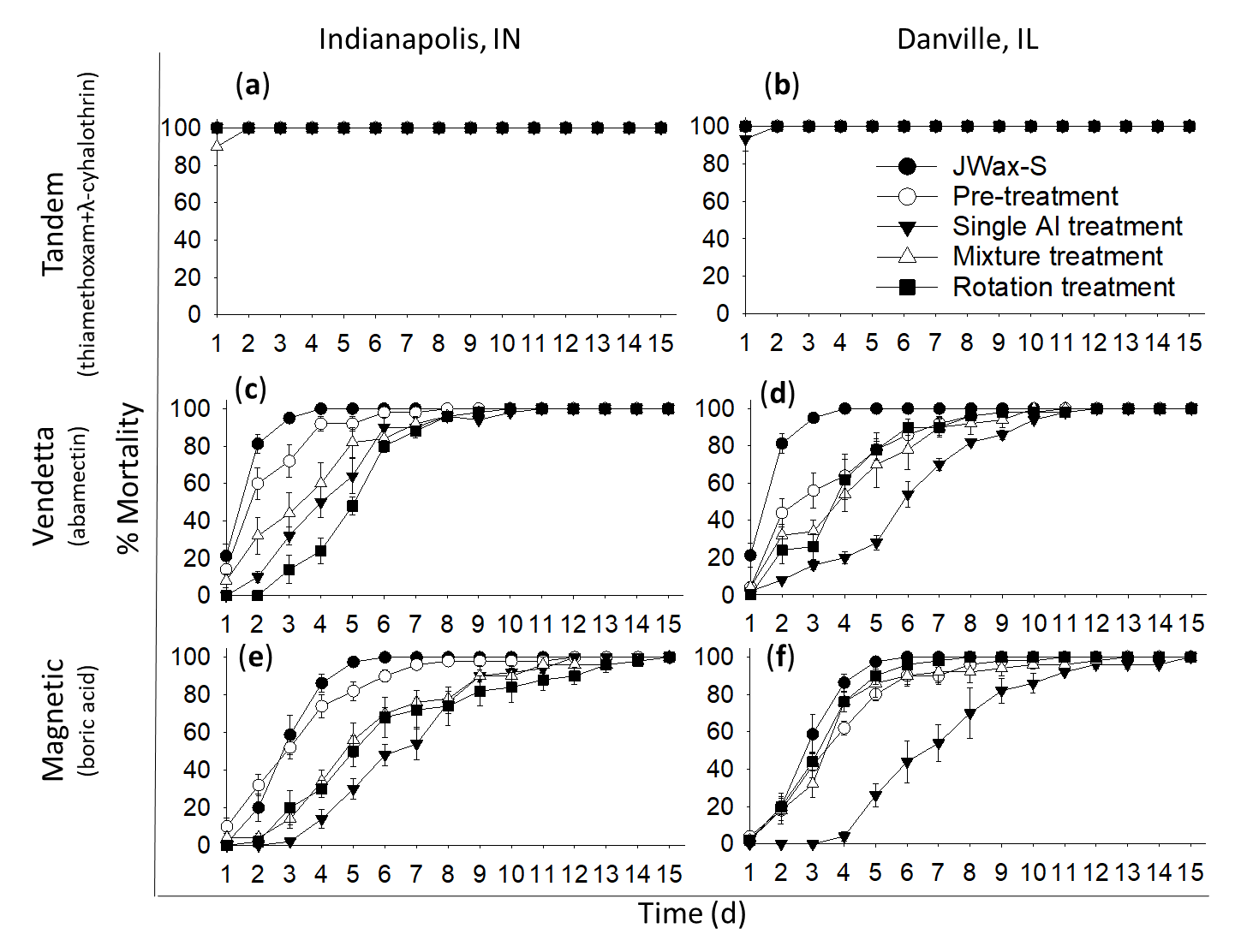


**Fig. S2.** Assessment of (**a, b**) Tandem® spray (**c, d**) Vendetta^TM^ gel bait and (**e, f**) Magnetic^TM^ gel bait using no-choice assays. Cockroach strains tested included the JWax-S susceptible lab strain of *B. germanica*, as well as pre- and post-treatment ﬁeld-collected strains from Danville, IL and Indianapolis, IN. A summary of resistance ratios (RR) at lethal time (LT_50_, LT_90_) and confidence intervals (CI), determined from these time-mortality data, calculated using the Robertson et al. (2008) statistical analysis method, is provided in **Table S2**. See **Table 1** for treatment details.


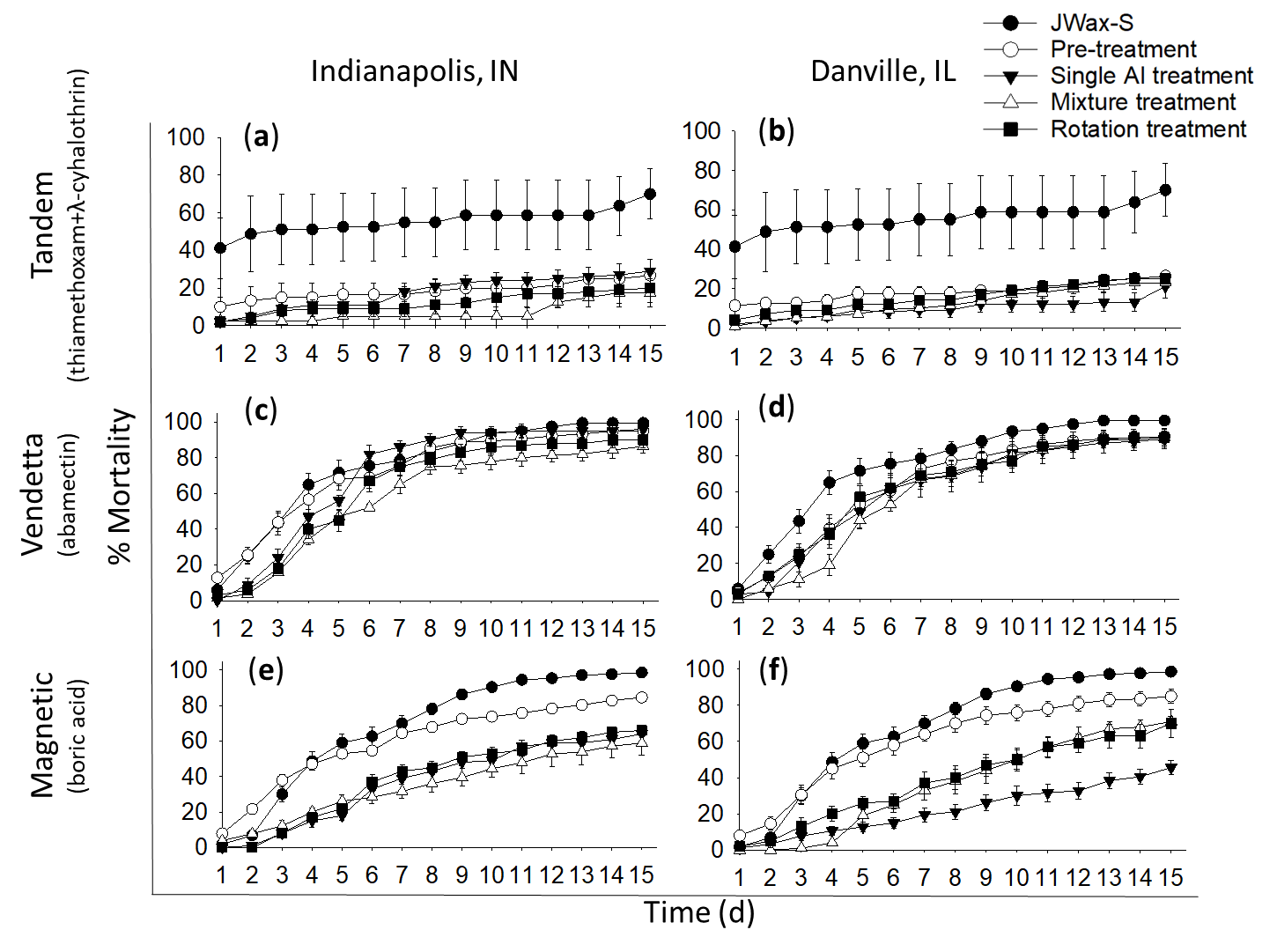


**Fig. S3.** Assessment of (**a, b**) Tandem® spray (**c, d**) Vendetta^TM^ gel bait, (**e, f**) Magnetic^TM^ gel bait using choice assays. Cockroach strains tested included the JWax-S susceptible lab strain of *B. germanica*, as well as pre- and post-treatment ﬁeld-collected strains from Indianapolis, IN (Left) and Danville, IL (Right). A summary of resistance ratios (RR) at lethal time (LT_50_, LT_90_) and confidence intervals (CI), determined from these time-mortality data, calculated using the Robertson et al. (2008) statistical analysis method, is provided in **Table S3**. See **Table 1** for treatment details.
